# Acetylcholine release and inhibitory interneuron activity in hippocampal CA1

**DOI:** 10.3389/fnsyn.2014.00020

**Published:** 2014-09-16

**Authors:** A. Rory McQuiston

**Affiliations:** Department of Anatomy and Neurobiology, Virginia Commonwealth UniversityRichmond, VA, USA

**Keywords:** hippocampus, acetylcholine, muscarinic, nicotinic, inhibitory interneuron

## Abstract

Acetylcholine release in the central nervous system (CNS) has an important role in attention, recall, and memory formation. One region influenced by acetylcholine is the hippocampus, which receives inputs from the medial septum and diagonal band of Broca complex (MS/DBB). Release of acetylcholine from the MS/DBB can directly affect several elements of the hippocampus including glutamatergic and GABAergic neurons, presynaptic terminals, postsynaptic receptors, and astrocytes. A significant portion of acetylcholine's effect likely results from the modulation of GABAergic inhibitory interneurons, which have crucial roles in controlling excitatory inputs, synaptic integration, rhythmic coordination of principal neurons, and outputs in the hippocampus. Acetylcholine affects interneuron function in large part by altering their membrane potential via muscarinic and nicotinic receptor activation. This minireview describes recent data from mouse hippocampus that investigated changes in CA1 interneuron membrane potentials following acetylcholine release. The interneuron subtypes affected, the receptor subtypes activated, and the potential outcome on hippocampal CA1 network function is discussed.

## Introduction

Acetylcholine is released throughout the mammalian central nervous system (CNS) where it impacts global brain function by affecting sleep-wake cycles, attention, and memory formation. One region of the brain heavily innervated by cholinergic afferents from the medial septum and diagonal band of Broca complex (MS/DBB) is the hippocampus (Dutar et al., 1995). Functionally, acetylcholine release in the hippocampus has been proposed to aid in the formation or retrieval of memories depending on the extracellular concentration of acetylcholine (Power et al., 2003; Hasselmo and Giocomo, 2006; Kenney and Gould, 2008; Deiana et al., 2011; Hasselmo and Sarter, 2011; Easton et al., 2012; Blake et al., 2014). The mechanism by which MS/DBB cholinergic terminals affect hippocampal network function is through the activation of both muscarinic and nicotinic receptors located on dendrites, cell bodies, and axon terminals of pyramidal neurons and inhibitory interneurons, as well as on astrocytes (Cobb and Davies, 2005; Teles-Grilo Ruivo and Mellor, 2013). Although acetylcholine affects multiple sites on several different cell types, a portion of its influence likely arises from its effects on interneuron function.

Inhibitory interneurons play a crucial role in information processing in the hippocampus. Interneurons are very diverse in anatomical structure and presumed function (Freund and Buzsaki, 1996; Klausberger and Somogyi, 2008). Depending on the interneuron subtype and where it innervates the pyramidal cell, an individual interneuron can completely block activity in a dendrite, change action potential firing phase at the soma, or completely prevent action potential firing at the pyramidal cell body (Miles et al., 1996; Larkum et al., 1999). At the network level, interneurons contribute to the generation of synchronous activity among populations of principal neurons at a variety of behaviorally relevant frequencies (Buzsaki, 2002; Buzsaki and Wang, 2012). Given the significant impact individual interneurons have on neuronal network function, it is probable that a considerable proportion of acetylcholine's influence on hippocampal activity arises through interneuron modulation. Although cholinergic receptors have been shown to affect inhibitory presynaptic terminals (Behrends and Ten Bruggencate, 1993; Tang et al., 2011) and interneuron excitability (McQuiston and Madison, 1999b; Griguoli et al., 2009; Cea-Del Rio et al., 2010, 2011), this minireview will limit its focus to recent studies that have investigated the effect of acetylcholine release on changes in interneuron membrane potential, specifically in hippocampal CA1.

## MS/DBB cholinergic neuron activity and acetylcholine release in hippocampal CA1

The impact that acetylcholine release has in hippocampal CA1 and the extent to which different interneuron subtypes are affected will depend on the specific location and density of cholinergic axon terminals as well as its inactivating enzyme, acetylcholinesterase. Notably, both cholinergic fibers and acetylcholinesterase have been shown to be differentially distributed across layers in hippocampal CA1. In mouse, cholinergic fibers were shown to be evenly distributed except for two bands of higher density in the stratum pyamidale (SP) and at the border between the stratum radiatum (SR) and stratum lacunosum-moleculare (SLM) (Aznavour et al., 2002). In rat, similar higher density bands were observed in the SP and at the border of SR and SLM. However, compared to the stratum oriens (SO), lower densities were seen in the SR and even lower densities in SLM (SO > SR > SLM) (Aznavour et al., 2002). The distribution of acetylcholinesterase in hippocampal CA1 complements that of cholinergic input, with higher densities observed between SP and SO as well as another peak in SLM near the border with SR (Storm-Mathisen, 1970). Consistent with these anatomical data, measurements of increased acetylcholine release during theta rhythms have shown that acetylcholine concentrations were highest near the stratum pyramidale (Zhang et al., 2010). This differential distribution of cholinergic fibers and extracellular acetylcholine levels is particularly important when considering that not all cholinergic terminals in the hippocampus appear to transmit acetylcholine synaptically. In both the hippocampus and neocortex, 85–93% of cholinergic axon terminals were estimated to have no postsynaptic specialization and thus the majority of cholinergic terminals were proposed to transmit acetylcholine by volume or non-synaptic transmission (Umbriaco et al., 1994, 1995). However, other groups have estimated that the majority of cholinergic terminals (66–67%) in the neocortex make classical synaptic connections (Smiley et al., 1997; Turrini et al., 2001). Regardless of this discrepancy, a significant portion of terminals appear to release acetylcholine into the extracellular space in a paracrine-like manner. This requires terminally released acetylcholine to diffuse significant distances past acetylcholinesterase to bind to receptors on postsynaptic elements. Thus, regions or layers with favorable densities of cholinergic terminals (higher) and/or acetylcholinesterase (lower) may result in larger extracellular concentrations of acetylcholine that may be more effective at transmitting acetylcholine through volume transmission. Furthermore, it is possible that there is a subset of terminals that are more active, have a higher probability of release, or may release more neurotransmitter. These terminals may be more effective at mediating volume transmission and influencing nearby inhibitory interneurons.

Acetylcholine release from cholinergic terminals will depend on the activity of the cholinergic neurons in the MS/DBB. However, the firing patterns of MS/DBB cholinergic neurons reported in the literature have shown some variability (Barrenechea et al., 1995; Brazhnik and Fox, 1997, 1999; Simon et al., 2006). A small number of anatomically identified MS/DBB cholinergic neurons recorded in awake restrained rodents have been reported to have low irregular firing rates (<2 Hz) (Simon et al., 2006). In contrast, anatomically unidentified neurons with action potential waveforms consistent with MS/DBB cholinergic neurons have been reported to fire at rates up to 30 Hz (Brazhnik and Fox, 1999). Thus, it remains unclear which rates best describe the firing patterns of cholinergic neurons in the MS/DBB or whether they fall along a wide continuum. Nevertheless, potential differences in the firing frequency or the duration of activity of cholinergic neurons could have variable effects on different interneuron subtypes through local differences in acetylcholine concentrations.

## Effects of muscarinic receptor activation on hippocampal CA1 inhibitory interneurons

Disruption of the MS/DBB cholinergic function by systemic blockade of muscarinic receptors or direct injection of muscarinic receptor antagonists into the hippocampus can impair memory and the encoding of spatial information (Blokland et al., 1992; Atri et al., 2004; Hasselmo, 2006). A potential role for inhibitory interneurons in muscarinic receptor modulation of hippocampal function was initially based on observations that the exogenous application of cholinergic agonists resulted in an increase in spontaneous inhibitory postsynaptic currents (sIPSCs) in CA1 pyramidal neurons (Pitler and Alger, 1992). These data indirectly suggested that a subset of inhibitory interneurons may be depolarized by muscarinic receptor activation and were subsequently confirmed by direct recordings (Parra et al., 1998; McQuiston and Madison, 1999a). However, not all interneurons responded to muscarinic receptor activation by depolarizing. Some interneurons were hyperpolarized or exhibited biphasic responses, and some failed to respond to the exogenous application of muscarinic agonist (Parra et al., 1998; McQuiston and Madison, 1999a). Moreover, each muscarinic response type could not be correlated with a morphological subtype of interneuron. These findings were further complicated by the observation that muscarinic receptors can inhibit the release of GABA from a subset of perisomatic inhibitory interneurons (Behrends and Ten Bruggencate, 1993; Fukudome et al., 2004; Szabo et al., 2010) and muscarinic receptor activation can increase interneuron excitability through the generation of after depolarizations (McQuiston and Madison, 1999b; Lawrence et al., 2006). Thus, the impact that acetylcholine release has on the interneuron population is complex and results in the recruitment of some interneurons while inhibiting others.

## Activation of muscarinic receptors in hippocampal CA1 interneurons following acetylcholine release

Although cholinergic muscarinic synaptic responses were first measured in CA1 pyramidal neurons in 1983 (Cole and Nicoll, 1983), it was not until 2006 that muscarinic responses to electrically evoked acetylcholine release were measured in hippocampal CA1 inhibitory interneurons (Widmer et al., 2006). This study showed that terminally released acetylcholine had divergent effects on different interneuron subtypes. Interneurons could respond by depolarizing, hyperpolarizing, or with biphasic responses. Overall, the majority of responding interneurons produced depolarizations (64%) whereas hyperpolarizations were infrequently observed (13%) (Widmer et al., 2006). Moreover, like previous studies using exogenous application of muscarinic agonists (Parra et al., 1998; McQuiston and Madison, 1999a), the different electrically evoked muscarinic response types could not be correlated with specific interneuron anatomical subtypes (Widmer et al., 2006). These findings have been recently confirmed by optogenetic studies using evoked release in response to light-activation (Nagode et al., 2011; Bell et al., 2013). However, in one of these optogenetic studies, interneurons responding with biphasic (25%), hyperpolarizing (35%), and depolarizing (40%) muscarinic responses were more equally distributed among the different response types (Bell et al., 2013). Importantly, optogenetically released acetylcholine predominantly produced muscarinic responses (80%) vs. nicotinic responses (17%). The remaining 3% of responding interneurons had both muscarinic and nicotinic responses. Furthermore, the muscarinic hyperpolarizations were mediated by the activation of M_4_ receptors whereas the depolarizations were likely produced by M_3_ receptor activation (Bell et al., 2013). Similar to the electrical stimulation studies, muscarinic response type could not be correlated with anatomical interneuron subtypes. Importantly, both studies showed that perisomatically projecting interneurons (likely parvalbumin-expressing basket cells) could respond to acetylcholine release with any one of the three muscarinic response types (Widmer et al., 2006; Bell et al., 2013). In different optogenetic studies, CA1 interneuron membrane potential was indirectly assessed by measuring sIPSC frequency in CA1 pyramidal neurons (Nagode et al., 2011, 2014). Optogenetically released acetylcholine resulted in an increase in large amplitude sIPSCs with frequencies that fell within the theta bandwidth (4–12 Hz) (Nagode et al., 2011). Importantly, this increase in sIPSCs could be inhibited by endocannabinoids suggesting that they resulted from the activation of cholecystokinin positive interneurons (Nagode et al., 2011). Furthermore, the sIPSCs were not affected by optogenetic suppression of parvalbumin positive cells, suggesting they did not arise from the activation of parvalbumin basket cells, axo-axonic, bistratified or oriens-lacunosum-moleculare interneurons (Nagode et al., 2014). These findings are consistent with synaptic stimulation studies, which recorded from an interneuron with cholecystokinin basket cell morphology that produced a biphasic response to acetylcholine release (Widmer et al., 2006). Therefore, based on effects on the membrane potential alone, endogenously activated muscarinic receptors on hippocampal CA1 interneurons will have complex effects on network function (see Table 1).

**Table 1:** **Cholinergic responses vary in similar and different anatomical interneuron subtypes**.

**Interneuron axonal arborization**	**Muscarinic depol.**	**Muscarinic hyperpol.**	**Muscarinic biphasic**	**Nicotinic α7**	**Nicotinic α4β2**	**Nicotinic α2**
Perisomatic SP	Agonist: Parra et al., 1998; McQuiston and Madison, 1999a	Agonist: McQuiston and Madison, 1999a	Agonist: McQuiston and Madison, 1999a	Agonist: McQuiston and Madison, 1999c; Buhler and Dunwiddie, 2001	Agonist: Not identified	Agonist: Not identified
	Synaptic: Widmer et al., 2006; Nagode et al., 2011, 2014; Bell et al., 2013	Synaptic: Widmer et al., 2006; Bell et al., 2013	Synaptic: Widmer et al., 2006; Bell et al., 2013	Synaptic: Not identified	Synaptic: Not identified	Synaptic: Not observed
Proximal dendritic SR or SO	Agonist: Parra et al., 1998; McQuiston and Madison, 1999a	Agonist: McQuiston and Madison, 1999a	Agonist: McQuiston and Madison, 1999a	Agonist: McQuiston and Madison, 1999c; Buhler and Dunwiddie, 2001	Agonist: Not identified	Agonist: Not identified
	Synaptic: Widmer et al., 2006; Bell et al., 2013	Synaptic: Widmer et al., 2006; Bell et al., 2013	Synaptic: Widmer et al., 2006; Bell et al., 2013	Synaptic: Not identified	Synaptic: Bell et al., 2011	Synaptic: Not observed
Distal dendritic SLM	Agonist: Parra et al., 1998; McQuiston and Madison, 1999a	Agonist: Parra et al., 1998; McQuiston and Madison, 1999a	Agonist: McQuiston and Madison, 1999a	Agonist: McQuiston and Madison, 1999c; Buhler and Dunwiddie, 2001; Griguoli et al., 2009	Agonist: Griguoli et al., 2009	Agonist: McQuiston and Madison, 1999c; Griguoli et al., 2009
	Synaptic: Widmer et al., 2006; Bell et al., 2013	Synaptic: Widmer et al., 2006; Bell et al., 2013	Synaptic: Bell et al., 2013	Synaptic: Not identified	Synaptic: Bell et al., 2011	Synaptic: Not observed

Cholinergic responsive interneurons are categorized based on the anatomical location of their axons (left column). References are reported for cholinergic response types observed in each class of interneuron. Agonist refers to responses elicited by exogenous agonist application. Stimulation refers to endogenous acetylcholine responses elicited electrically or optogenetically. Not identified—indicates that such a response type has not been observed in that class of interneuron. Not observed—indicates that no such response type has been observed in any interneuron class.

Although different muscarinic response types were almost uniformly observed in CA1 interneurons, not all response types were as easily evoked by optogenetic stimulation (Bell et al., 2013). Consistent with some *in vivo* recordings (Brazhnik and Fox, 1999), acetylcholine released from MS/DBB cholinergic terminals by blue light flashes delivered at 20 Hz was capable of producing each response type in hippocampal CA1 interneurons (Bell et al., 2013). However, the number of flashes affected the probability of observing a particular response type. In hyperpolarizing interneurons, 10 flashes were sufficient (91% of hyperpolarizing interneurons) to observe a response. In contrast, 10 flashes were not sufficient to produce a response in the majority of depolarizing interneurons (58%). Similarly, the depolarizing phase could not be observed in the majority of biphasic interneurons (55%) when only 10 stimuli were delivered. Therefore, muscarinic hyperpolarizations may require less presynaptic MS/DBB cholinergic activity compared to depolarizing responses in hippocampal CA1 interneurons. It may be that suppression of interneuron excitability will be the predominant effect in response to low levels of MS/DBB cholinergic activity.

## Effects of nicotinic receptor activation on hippocampal CA1 interneurons

Activation of nicotinic receptors in the hippocampus has a significant impact on physiological and pathophysiological memory formation (Levin, 2002; Levin et al., 2002, 2009; Buccafusco et al., 2005; Davis and Gould, 2006, 2009; Nott and Levin, 2006; Davis et al., 2007). Of the 11 different nicotinic receptor subunits found in the mammalian CNS, 9 have been reported to be expressed in hippocampal CA1 neurons (Sudweeks and Yakel, 2000). Using exogenous application of nicotinic agonists, functional nicotinic receptors that contain α7 (Alkondon et al., 1997; Jones and Yakel, 1997; Frazier et al., 1998b; McQuiston and Madison, 1999c), α4β2 (McQuiston and Madison, 1999c; Sudweeks and Yakel, 2000), or α2 subunits (McQuiston and Madison, 1999c; Sudweeks and Yakel, 2000; Jia et al., 2009) have been observed in hippocampal CA1 interneurons. Although hippocampal interneurons appeared to express a diverse collection nicotinic receptor subtypes, α7 containing receptors were more frequently observed and produced larger responses (McQuiston and Madison, 1999c; Sudweeks and Yakel, 2000). Indeed, α7 nicotinic receptors in the hippocampus have been associated with memory formation (Levin, 2002; Levin et al., 2002; Nott and Levin, 2006) and their dysfunction may play a role in some forms of schizophrenia (Freedman et al., 1994; Leonard et al., 1996; Adler et al., 1998). However, despite their lower expression levels, the α4β2 containing nicotinic receptors have been reported to play a significant role in memory formation (Davis and Gould, 2006; Davis et al., 2007) and in hippocampal-dependent nicotine addiction (Perry et al., 1999; Davis and Gould, 2009). α4β2 containing receptors have also been correlated with cognitive deficits associated with aging and Alzheimer's disease (Kellar et al., 1987; Wu et al., 2004; Gahring et al., 2005). To fully understand the role that different nicotinic subunits play in the hippocampus, the effect of endogenously released acetylcholine on individual hippocampal cells and the hippocampal network has begun to be investigated.

## Activation of nicotinic receptors in hippocampal CA1 interneurons following acetylcholine release

Acetylcholine release from MS/DBB cholinergic terminals in hippocampal CA1 has been demonstrated to activate nicotinic receptors on interneurons (Alkondon et al., 1998; Frazier et al., 1998a; Stone, 2007). Nicotinic excitatory postsynaptic currents (EPSCs) were first observed using electrical stimulation and whole cell patch clamping in acute rat brain slices. These nicotinic EPSCs had fast kinetics and were blocked by α7 nicotinic receptor antagonists (Alkondon et al., 1998; Frazier et al., 1998a), consistent with studies that applied nicotinic receptor agonists directly onto interneuron cell bodies (Alkondon et al., 1997; Jones and Yakel, 1997; Frazier et al., 1998b; McQuiston and Madison, 1999c). However, more recent optogenetic studies in mouse brain slices were not able to reproduce these earlier observations (Bell et al., 2011). Instead, optogenetically released acetylcholine primarily activated nicotinic receptors that contained α4β2 subunits. Furthermore, the α4β2 responses were mostly subthreshold and had very slow kinetics. These data were suggestive of acetylcholine diffusing a significant distance before binding to the α4β2 containing nicotinic receptors (McQuiston and Madison, 1999c; Bennett et al., 2012), consistent with volume or non-synaptic transmission (Vizi et al., 2010). Although these small nicotinic responses could temporally summate, their ability to excite interneurons was limited through muscarinic presynaptic inhibition. Because the nicotinic responses were mostly subthreshold, nicotinic transmission onto CA1 interneurons may be primarily modulatory in nature. The optogenetic studies also examined the nicotinic responses using voltage-sensitive dye (VSD) imaging. The nicotinic VSD signals were completely blocked by the α4β2 receptor antagonist DHβE and were found to be significantly larger in the distal dendritic region of CA1 pyramidal neurons, which overlaps with inputs from the entorhinal cortex and nucleus reuniens of the thalamus (Bell et al., 2011). Importantly, because the VSD stains all elements of the tissue, the VSD data suggest that α4β2 containing nicotinic receptors are the most prevalent receptor that mediates depolarizing nicotinic responses in mouse hippocampal CA1. Notably, nicotinic responses could be produced by a single flash of light (Bell et al., 2011) suggesting that acetylcholine release from MS/DBB cholinergic terminals may help recruit interneurons via nicotinic receptor activation before they are affected by muscarinic receptor activation.

## Effects of acetylcholine release on hippocampal CA1 network function from the perspective of the interneuron membrane potential

Because CA1 inhibitory interneuron membrane potentials can be differentially modulated by both muscarinic and nicotinic receptor activation following acetylcholine release, the consequential effect on network function is undoubtedly complex. Muscarinic receptor activation can result in varying and opposing effects, even within the same interneuron (see Table 1). Unfortunately, our understanding of how each subtype of interneuron can be affected by muscarinic or nicotinic receptor activation remains incomplete. Nevertheless, the number of stimuli required to produce each type of response varied in a consistent manner. Nicotinic responses were most easily evoked requiring the fewest number of stimuli (Bell et al., 2011) whereas depolarizing muscarinic responses were the most difficult to produce requiring the largest number stimuli (Bell et al., 2013). Therefore, it can be hypothesized that low levels of MS/DBB cholinergic neuron activity and lower concentrations of extracellular acetylcholine favor the activation nicotinic receptors or a muscarinic hyperpolarization in specific subsets of CA1 interneurons.

**Figure 1:**
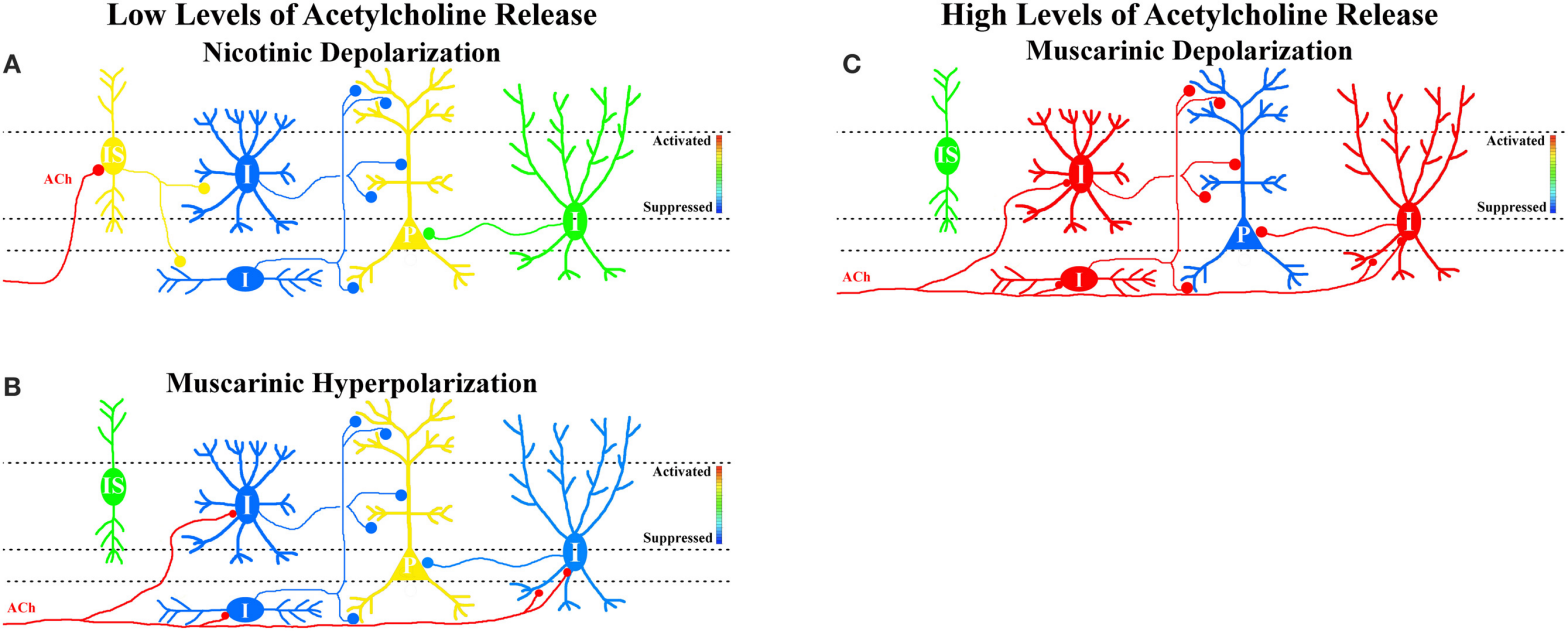
**Hypothesis that MS/DBB cholinergic inputs either suppress or activate interneuron networks in hippocampal CA1 depending on cholinergic neuron activity. (A)** Low levels of MS/DBB cholinergic activity preferentially activate subsets of interneurons through the activation of nicotinic receptors. We postulate that nicotinic-driven interneurons are interneurons-selective interneurons (IS, yellow—activation) that specifically inhibit other interneurons (blue). Increasing their activity results in disinhibition of pyramidal neurons (P, yellow—activation, and increased output). **(B)** Low levels of MS/DBB cholinergic activity also hyperpolarize subsets of interneurons through the activation of muscarinic receptors (I, blue—suppression) resulting in disinhibition of pyramidal neurons (P, yellow—activation, and increased output). **(C)** Increasing cholinergic neuron activity causes subsets of interneurons to be depolarized by muscarinic receptor activation (I, red—activation, and increased synaptic inhibition) resulting in suppression of pyramidal neurons (P, blue—suppressed output).

Because muscarinic hyperpolarization of CA1 interneurons requires less presynaptic cholinergic activity, disinhibition (indirect activation) of hippocampal CA1 pyramidal cells may be favored during low levels of MS/DBB cholinergic activity (Figure 1B). Furthermore, postulating that nicotinic responses preferentially affect interneurons that selectively inhibit other interneurons (interneuron-selective or IS), nicotinic receptor activation may also result in disinhibition of CA1 pyramidal neurons (Figure 1A). Together, low levels of MS/DBB cholinergic activity would favor a net disinhibition of hippocampal CA1 permitting higher probability of output from CA1 pyramidal neurons. Increased output from CA1 may result in the facilitation of recall and memory consolidation in other areas of the CNS as is thought to occur during slow wave sleep (Gais and Born, 2004; Hasselmo and McGaughy, 2004). In contrast, higher levels of MS/DBB cholinergic neuron activity coupled to higher extracellular concentrations of acetylcholine will subsequently recruit different subsets of interneurons that respond via muscarinic depolarizations. Some of these depolarizing interneurons may impose rhythmic inhibition of CA1 pyramidal neurons at theta frequencies (Nagode et al., 2011, 2014), a network rhythm observed during higher levels of acetylcholine release (Zhang et al., 2010). This would result in inhibition of hippocampal CA1 pyramidal neuron output (partly rhythmic) while facilitating synaptic integration within hippocampal CA1 pyramidal cell dendrites through cholinergic effects on glutamatergic receptors and dendritic function (Figure 1C) (Tsubokawa and Ross, 1997; Tsubokawa, 2000; Fernandez De Sevilla and Buno, 2010; Giessel and Sabatini, 2010). Indeed, such a dynamic role for acetylcholine concentrations in learning and memory formation has been previously proposed (Hasselmo, 2006; Hasselmo and Giocomo, 2006; Giocomo and Hasselmo, 2007; Hasselmo and Sarter, 2011). In this scheme, lower acetylcholine concentrations permit intrahippocampal (Schaffer collaterals) synaptic interactions to dominate thus increasing hippocampal CA1 output and memory retrieval, whereas higher acetylcholine concentrations favor processing of inputs from outside the hippocampus permitting the transient formation of memories in hippocampal CA1. Therefore, the combined effect of acetylcholine release on glutamatergic inputs and interneuron function may play important roles in tuning the hippocampal CA1 network for recall or to form new memories.

### Conflict of interest statement

The author declares that the research was conducted in the absence of any commercial or financial relationships that could be construed as a potential conflict of interest.
